# Psychosis Secondary to Neurocysticercosis: Case Report and Narrative Review

**DOI:** 10.1192/j.eurpsy.2026.12202

**Published:** 2026-07-31

**Authors:** G. Afonso, M. R. Pinto, R. L. de Dios, J. de Sousa, J. Mesquita

**Affiliations:** Department of Psychiatry, Braga Local Health Unit, Braga, Portugal

## Abstract

**Introduction:**

Neurocysticercosis (NCC) is an infection of the central nervous system (CNS) caused by the larval stage of Taenia solium. It is the most common parasitic disease of the CNS (Del Brutto OH. Scientific World Journal 2012; 159821). Although more prevalent in developing countries, the progressive increase in migration and international travel has raised its prevalence in developed countries. NCC predominantly presents with neurological symptoms but may also be associated with psychiatric manifestations, including psychosis.

**Objectives:**

To characterise the main features of a psychotic episode secondary to NCC, including its most frequent clinical manifestations and the most appropriate treatment approaches.

**Methods:**

Relevant and recent scientific articles were selected from PubMed®. The theoretical evidence derived from this review was contextualised with a clinical case of an 86-year-old woman with no previous psychiatric history, who developed a psychotic episode secondary to NCC.

**Results:**

Psychosis is a rare manifestation of NCC, occurring in up to 5% of cases (Mahajan et al. J Assoc Physicians India 2004; 52 663-5). Psychiatric symptoms in NCC are associated with increased cerebrospinal fluid pressure and inflammatory lesions of the brain parenchyma caused by cystic formations (Anjana et al. Arch Ment Health 2020; 21 55-58). In this context, psychotic episodes present with delusional ideation, disorganised speech, psychomotor agitation with aggressive behaviour, and visual hallucinations (El-Kady et al. Neuropsychiatr Dis Treat 2021; 17 1599-1610). Although not exclusive to psychosis, the most striking neuropsychiatric manifestations of NCC have been reported when cysts lodge in the prefrontal and occipital lobes (Goyal et al. Neurol India 2023; 71 228-232). Treatment should combine the use of antipsychotics and, in acute NCC, antiparasitic drugs and corticosteroids.

**Conclusions:**

As described in the literature, the patient presented with a psychotic episode characterised by auditory and olfactory hallucinations, paranoid and persecutory delusional ideation, disorganised speech, and psychomotor agitation. Brain MRI revealed cystic lesions in the bilateral frontal lobes and in the right caudate nucleus. Treatment with the antipsychotic paliperidone (6 mg/day) led to complete remission of hallucinatory activity and partial remission of delusional ideation, now encapsulated.

**Disclosure of Interest:**

None Declared

